# Determinants of aggregate anthropometric failure among children under-five years in Ethiopia: Application of multilevel mixed-effects negative binomial regression modeling

**DOI:** 10.1371/journal.pgph.0003305

**Published:** 2024-06-04

**Authors:** Biniyam Sahiledengle, Lillian Mwanri

**Affiliations:** 1 Department of Public Health, Madda Walabu University Goba Referral Hospital, Bale-Goba, Ethiopia; 2 Research Centre for Public Health, Equity and Human Flourishing, Torrens University Australia, Adelaide Campus, SA 5000, Adelaide, Australia; WorldFish, MALAYSIA

## Abstract

Undernutrition significantly contributes to failure to thrive in children under five, with those experiencing multiple forms of malnutrition facing the highest risks of morbidity and mortality. Conventional markers such as stunting, wasting, and underweight have received much attention but are insufficient to identify multiple types of malnutrition, prompting the development of the Composite Index of Anthropometric Failure (CIAF) and the Composite Index of Severe Anthropometric Failure (CISAF) as an aggregate indicators. This study aimed to identify factors associated with CIAF and CISAF among Ethiopian children aged 0–59 months using data from the 2019 Ethiopia Mini Demographic and Health Survey. The study included a weighted sample of 5,259 children and used multilevel mixed-effects negative binomial regression modeling to identify determinants of CIAF and CISAF. The result showed higher incidence-rate ratio (IRR) of CIAF in male children (adjusted IRR = 1.27; 95% CI = 1.13–1.42), children aged 12–24 months (aIRR = 2.01, 95%CI: 1.63–2.48), and 24–59 months (aIRR = 2.36, 95%CI: 1.91–2.92), those from households with multiple under-five children (aIRR = 1.16, 95%CI: 1.01–1.33), poorer households (aIRR = 1.48; 95%CI: 1.02–2.15), and those who lived in houses with an earthen floor (aIRR = 1.37, 95%CI: 1.03–1.82). Similarly, the factors positively associated with CISAF among children aged 0–59 months were male children (aIRR = 1.47, 95% CI = 1.21–1.79), age group 6–11 months (aIRR = 2.30, 95%CI: 1.40–3.78), age group 12–24 months (aIRR = 3.76, 95%CI: 2.40–5.88), age group 25–59 months (aIRR = 4.23, 95%CI: 2.79–6.39), children from households living with two and more under-five children (aIRR = 1.27, 95%CI:1.01–1.59), and children from poorer households (aIRR = 1.93, 95% CI = 1.02–3.67). Children were more likely to suffer from multiple anthropometric failures if they were: aged 6–23 months, aged 24–59 months, male sex, living in households with multiple under-five children, and living in households with poor environments. These findings underscore the need to employ a wide range of strategies to effectively intervene in multiple anthropometric failures in under-five children.

## Background

Childhood undernutrition, including stunting, wasting, and underweight remains a significant contributor to the global burden of disease. Globally, in the year 2020, an estimated 149 million (22%) children under five years of age were stunted, and 45 million (7%) were wasted [1]. The global burden of childhood undernutrition is concentrated in low-income and lower-middle-income countries. Sub-Saharan African (SSA) countries were the most affected region and two out of five stunted children and over one-quarter of all wasted children under five live in this region [2]. In the Eastern African region where Ethiopia is situated, childhood undernutrition continues to be a rampant public health concern [3].

In Ethiopia, despite the significant impact of childhood undernutrition on child survival and development, recent progress has been gradual to enable the nation to achieve the 2030 Sustainable Development Goal (SDG) target. Between 2005 and 2019, for example, the prevalence of childhood stunting decreased from 51% to 37%, wasting decreased from 12% to 7%, and underweight decreased from 33% to 21% [4,5]. Previous studies have suggested that sex of the child, wealth status, childhood illness, household food insecurity, poor sanitation, short birth interval, lack of exclusive breastfeeding, low maternal education, and unhealthy environment are associated with child undernutrition [6–10].

Prior studies in Ethiopia have focused on stunting, wasting, and underweight using traditional nutritional indicators [11–15], others concentrate on socioeconomic inequality [16,17], spatial analysis of undernutrition [18–20], and double-burden of malnutrition [21,22]. However, children who are underweight may experience stunting and/or wasting, and some children may suffer all three anthropometric failures simultaneously. Therefore, none of these conventional nutritional indicators are able to precisely characterize the overall burden of undernutrition among children under the age of five. To address this issue, the composite index for anthropometric failure (CIAF) or the composite index of severe anthropometric failure (CISAF), a multidimensional single index was introduced by Svedburg and Nandy in 2000 [23,24] and Vollmer and colleagues in 2017 [25]. The CIAF/CISAF incorporates all three types of undernutrition, yielding a single aggregate value of all undernourished children in a population.

The CIAF/CISAF provides a deeper understanding of the extent of severe childhood undernutrition in resource-limited settings, which is computed by aggregating different categories of one or more forms of anthropometric failures such as: wasting only, wasting and underweight, wasting, stunting and underweight, stunting and underweight, stunting only and underweight only. The CIAF is one of the newly proposed indices which has been utilized in prior studies [26–29] and has been strongly recommended as an alternative measure of malnutrition. This index aids in identifying multiple nutritional deficiencies, serving as an aggregate indicator [30]. In Asia, CISAF has been successfully used to demonstrate the prevalence of severe undernutrition among the children under-five. For example in Bangladesh CISAF was 11.0% (rural 11.5% vs. urban 9.6%) [31], indicative of severe undernutrition in this nation, especially in children residing in rural settings.

Despite the profound benefit of using CIAF and/or CISAF, there is a dearth of evidence about the use of these indicators in SSA, and studies using aggregate anthropometric failure to identify factors associated with nutritional deficit in Ethiopia are scant [32–34]. Therefore, this study aimed to investigate factors associated with CIAF among children under the age of five in Ethiopia using a multilevel negative binomial regression modeling approach.

## Methods

### Data source

We used the Ethiopian Mini Demographic and Health Survey (EMDHS) 2019, which is a nationally representative survey (5).The 2019 EMDHS was designed using a multi-stage stratification, stratified by: (i) administrative area, (ii) urban and rural status, and (iii) equal probability systematic sampling. Information about the socioeconomic status, health, behaviors, and environmental conditions of children aged under 5 years and their parents with mothers aged 15–49 years were collected using standardized questionnaires [35].

### Measurement

**Fig 1:**
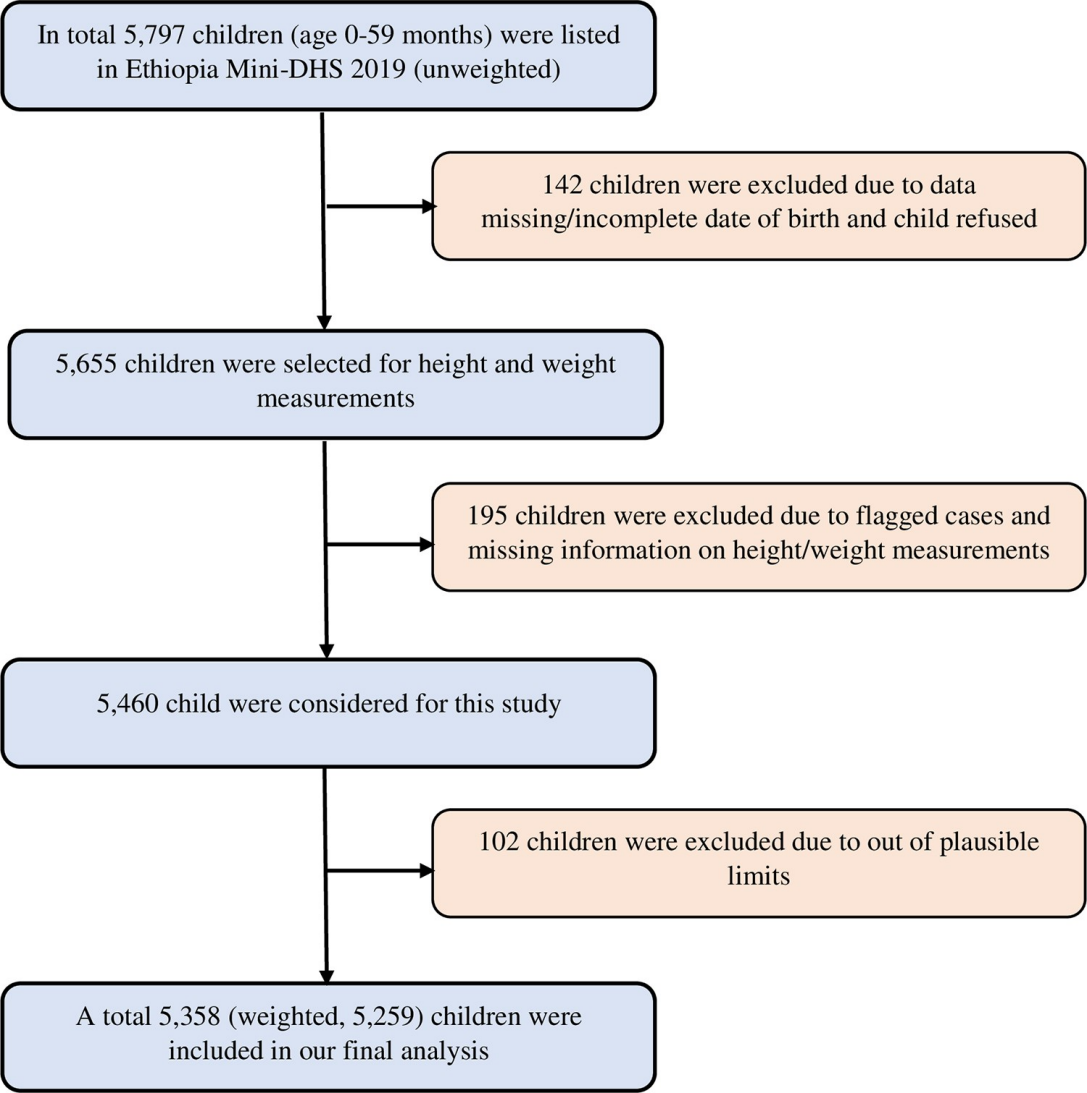
Sample size selection.

The 2019 EMDHS collected anthropometric data by measuring the height and weight of all children under age 5 in the selected households. Weight was measured with an electronic mother-infant scale (SECA 874 flat) designed for mobile use. A stadiometer was utilized to measure children under 24 months old while they were positioned in a recumbent posture on the measuring board. The instrument used for this purpose is referred to as an infantometer. This specialized tool is designed to accurately measure the length of infants and young children while they are lying down. Based on the 2006 World Health Organization (WHO) child growth standards, all anthropometric failure outcomes were generated [1]. Stunting was defined as a height-for-age Z scores (HAZ) of less than −2 standard deviations (SDs) of the median, wasting as a weight-for-height Z scores (WHZ) of less than −2 SDs, and underweight as a weight-for-age Z scores (WAZ) of less than −2 SDs. While children whose height-for-age Z-score, weight-for-height Z-score, and weight-for-age Z-score were below minus three standard deviations (-3SD) from the median are considered severely stunted, wasted, and underweight [1]. The 2019 EMDHS gathered anthropometric data from children under five (n = 5,279 for stunting, n = 5,408 for wasting, and n = 5,338 for underweight) [5]. Missing or implausible measures of children’s weight and height were excluded from our analytic sample. A total weighted sample of 5,259 children aged 0 to 59 months was included in this study (Fig 1).

### Outcome variables

The outcome variable for this analysis were the composite index of anthropometric failure (CIAF) and the composite index of severe anthropometric failure (CISAF) among children aged 0–59 months. Following a previous practice, nutritional indicators for children were divided into seven categories: (A) no failure; (B) wasting only; (C) wasting and underweight; (D) wasting, stunting, and underweight; (E) stunting and underweight; (F) stunting only; and (Y) underweight only. A child was considered undernourished, as measured by CIAF if they had any of the anthropometric failures (B-Y) [24,28] (S1 Table).

Based on an updated version of the CIAF by Vollmer et al. (2017), similar approaches were employed to compute the composite index of severe anthropometric failure (CISAF) [25]. CIAF considers a broader spectrum of anthropometric conditions, encompassing mild to moderate cases alongside severe cases. In contrast, CISAF focuses solely on severe cases of anthropometric failure, disregarding mild to moderate instances. A child was considered to be severely stunted and severely wasted and severely underweight if the z-scores of height-for-age, weight-for-height, and weight-for-age were below minus three standard deviations (i.e., HAZ < −3SD; WHZ < −3SD; WAZ < −3SD) below the respective median of the WHO reference population, respectively. Accordingly, severe nutritional indicators for under-5 children were categorized into seven groups: (A) no severe failure; (B) severe wasting only; (C) severe wasting and severe underweight; (D) severe wasting and severe stunting and severe underweight; (E) severe stunting and severe underweight; (F) severe stunting only; and (G) severe underweight only. Children without any anthropometric failure from B to G, that is, with moderate undernutrition and/or normal children were categorized as ‘no severe failure’. A child is considered having severe, as measured by CISAF if they had any of the anthropometric failures (B to G) (S2 Table).

Both the CIAF and CISAF are composite indices that aggregate multiple anthropometric indicators to assess nutritional status. Consequently, we constructed a count variable for CIAF and CISAF, ranging from 0 to 3, indicating the number of undernutrition problems experienced by each child. A score of 0 signifies no nutritional failure for CIAF or no severe failure in the case of CISAF, while a score of 1, 2, or 3 indicates additional manifestations of undernutrition experienced by a single child.

### Independent variables

The UNICEF conceptual framework of factors contributing to undernutrition guided our choice of variables [36]. In addition, the selection of the explanatory variables (i.e. individual and community-level factors) was made based on the availability of the variable in the DHS dataset. The individual-level factors included were the sex of the child, age, birth order, birth interval, number of under-five children, breastfeeding status, dietary diversity score, meal frequency, received vitamin A in the last 6 months, maternal age, maternal education, maternal postnatal visit, antenatal care, household wealth, household toilet facility, and household’s source of drinking water. Community-level factors include place of residence and region.

The wealth index was constructed using data collected through household surveys, including information on ownership of assets, housing conditions, and access to basic service. The principal components analysis (PCA) method was used by DHS to represent the household wealth index as a score of household assets. The DHS assigned each de jure household member scores after computing the index. Each household of participants was ranked by their score. The wealth index was categorized into five quintiles: the bottom 20% was classified as "poorest," the next 20% was classified as poorer, the next 20% as "middle," the next 20% as richer, and the top 20% as "richest."[37]. Maternal education was the mother’s highest level of education and was classified into four categories-no education, primary education, secondary education, and higher education. We defined the minimum dietary diversity of children aged 6–23 months based on a score ranging from 0 to 8, with one point assigned for consuming each of the following types of food during the previous day (over the previous 24 hours): The five groups should have come from a list of eight food groups: breast milk; grains, roots, and tubers; legumes and nuts; dairy products (milk, yogurt, and cheese); flesh foods (meat, fish, poultry, and liver/organ meat); eggs; vitamin A-rich fruits and vegetables; and other fruits and vegetables [5]. We referred improved sanitary in households as access to: a ventilated improved pit latrine, flushing piped sewer system, composting latrine, septic tank or pit latrine, or slab type pit latrine; and unimproved sanitation in households as using other methods of sanitation. For the source of drinking water, households were classified into improved water sources (having access to water from pipes, public faucets, protected wells or springs, boreholes, or bottled water) and unimproved otherwise [38].

### Data analysis

All analyses were conducted using Stata version 14.0 (Stata Corp, College Station, Texas, USA). Given the complexity of the two-stage sampling design of EMDHS, we accounted for clustering, sampling weights, and stratification provided by DHS in our analysis. The “*svy*” command was used in our analysis to account the complex survey design of EMDHS. A descriptive analysis was conducted to describe the child undernutrition. The CIAF/CISAF was a count variable ranging from 0 to 3, that indicated the manifestations of undernutrition a single child experienced. Our outcome variable was a positively skewed and followed the Poisson distribution. However, the assumption of Poison distribution was violated in our dataset, meaning that the sample variance was higher than the sample mean and a negative-binomial regression was used. The number of zeros was also examined in this study to decide whether to use zero-inflated poison models (ZIP) or zero-inflated negative binomial models (ZINB). Due to the hierarchical nature of DHS data (i.e., nested data) a multilevel mixed-effects negative binomial regression model was used to assess the association between CIAF/CISAF and individual and community-level determinants [39,40]. First, bivariate mixed-effects negative binomial regression analysis was performed to assess factors associated with CIAF/CISAF. A P-value less than 0.25 in the bivariate analysis was considered a candidate to be included in a multivariable mixed-effects negative binomial regression model. We use a manual method in pre-filter variable selection in bivariate multilevel analysis using p-value< 0.25. A higher threshold p-value like 0.25 may be used during variable selection to ensure that potentially important predictors are not overlooked at an early stage of model building [41,42]. Additionally, in large-scale studies or such as DHS datasets with substantial variability and complexity, employing a higher significance level in bivariate analysis may be warranted to account for the multitude of potential relationships and minimize the possibility of overlooking important findings. Consequently, four models were used. The primary model (empty or null model) was fitted without explanatory variables. The second model (individual-level factors), third model (community-level factors), and fourth model (full model) were adjusted for individual and community-level factors simultaneously. The reported likelihood-ratio test in our analysis showed that there was enough variability to favor a mixed-effects Poisson regression over a standard Poisson regression. Multicollinearity among the independent variables was tested using the Variance Inflation Factor (VIF) before their inclusion in the final regression model. The VIF of 5 has been recommended as the maximum level [43–45]. Adjusted incidence-rate ratios (aIRR), along with their corresponding 95% confidence intervals (CIs), were used to estimate the strength and direction of the association between the determinants and CIAF/CISAF. Variation between clusters were assessed by computing intra-class correlation coefficient (ICC), proportional change in variance (PCV) statistics and median incident rate ratio (MIRR). Finally, a model comparison was done using the deviance test, and the model with the lowest deviance was selected as the best-fit model [46]. In the final model, a p-value of < 0.05 was used to define statistical significance.

### Ethics statement

We used datasets provided by the Demographic Health Surveys programme and have not had any form of contact with the study participants. Informed consent for the present analysis was not necessary because secondary data analysis did not involve interaction with the participants. Ethical clearance for the Demographic Health Survey (DHS) was provided by the Ethiopia Health and Nutrition Research Institute (EHNRI) Review Board, the National Research Ethics Review Committee (NRERC) at the Ministry of Science and Technology, the Institutional Review Board of ICF International, and the CDC. Further information regarding the DHS data usage and ethical standards can be accessed online (https://dhsprogramcom/data/Access-Instructionscfm).

## Results

### Characteristics of the sample

Characteristics of the study participants are presented in Table 1. Of these, more than half (50.8%) of the children were males, 40.0% of the children were in the age category of 36–59 months and 26.3% of the children had minimum meal frequency. About 22.9% and 18.5% of the children did fall within the poorest and richest household index quintiles, respectively.

**Table 1:** Distribution of stunting, wasting, underweight, and composite index or anthropometric failure (CIAF) by categories of variables among children aged 0–59 months in Ethiopia, EMDHS 2019 (n = 5,259).

Characteristics	Weighted n (%)	Stunting, % (95%CI)	Wasting, % (95%CI)	Underweight, % (95%CI)
**Child-related factors**				
**Sex**				
Male	2,479 (50.8)	40.1 (38.2–42.1)	8.9 (7.8–10.1)	22.9 (21.3–24.6)
Female	2,399 (49.2)	33.5 (31.7–35.5)	5.4 (4.6–6.4)	19.1 (17.6–20.7)
**Age (months)**				
<6	514 (10.5)	17.0 (14.1–20.5)	9.4 (7.2–12.2)	9.7 (7.5–12.6)
6–11	465 (9.5)	27.8 (23.9–32.1)	6.2 (4.4–8.9)	18.1 (14.9–21.8)
12–24	1,109 (22.7)	33.1 (30.4–35.9)	7.5 (6.1–9.2)	19.0 (16.8–21.4)
25–35	838 (17.2)	46.7 (43.3–50.1)	8.4 (6.7–10.4)	26.1 (23.2–29.2)
36–59	1,952 (40.0)	42.2 (40.1–44.5)	6.1 (5.2–7.3)	23.8 (22.0–25.7)
**Number of under-five children**				
1	1,934 (39.6)	33.5 (31.5–35.7)	5.3 (4.4–6.4)	19.2 (17.5–21.0)
2+	2,945 (60.4)	39.1 (37.3–40.8)	8.4 (7.5–9.4)	22.2 (20.8–23.8)
**Birth order**				
1	1,039 (21.3)	34.9 (32.1–37.8)	4.3 (3.2–5.7)	17.6 (15.4–20.1)
2	877 (17.9)	33.7 (30.6–36.8)	7.7 (6.1–9.7)	18.3 (15.9–20.9)
3	697 (14.3)	37.1 (33.6–40.8)	6.1 (4.5–8.1)	18.1 (15.4–21.1)
4	577 (11.8)	36.6 (32.8–40.6)	6.3 (4.6–8.6)	20.8 (17.7–24.2)
5+	1,686 (34.6)	39.8 (37.5–42.2)	9.4 (8.1–10.9)	25.9 (23.9–28.1)
**Birth interval**				
< 24 months	798 (20.8)	42.7 (39.3–46.1)	9.3 (7.5–11.5)	27.7 (24.8–30.9)
≥ 24 months	3,030 (79.1)	35.8 (34.2–37.6)	7.6 (6.7–8.6)	20.4 (19.1–21.9)
**Vitamin A in last 6 months (n = 2,880)**				
Yes	1,571 (54.5)	30.8 (28.6–33.2)	8.7 (7.4–10.2)	18.2 (16.4–20.2)
No	1,309 (45.4)	36.2 (33.7–38.9)	6.8 (5.6–8.3)	20.2 (18.1–22.5)
**Currently breastfeeding**				
Yes	2,226 (45.6)	32.1 (30.2–34.1)	7.3 (6.3–8.5)	18.8 (17.3–20.5)
No	2,653 (54.4)	40.9 (39.1–42.8)	7.1 (6.2–8.1)	22.9 (21.4–24.6)
**Minimum meal frequency**				
Yes	1,385 (26.3)	32.9 (30.5–35.4)	6.1 (5.0–7.5)	19.6 (17.6–21.7)
No	3,873 (73.7)	38.1 (36.6–39.7)	7.3 (6.5–8.2)	21.9 (20.6–23.2)
**Minimum dietary diversity**				
Yes	227 (4.6)	25.2 (20.0–31.3)	4.6 (2.5–8.2)	14.5 (10.5–19.7)
No	4,652 (95.4)	37.5 (36.1–38.9)	7.3 (6.6–8.1)	21.4 (20.2–22.6)
**Maternal factors**				
**Age (years)**				
15–24	1,118 (22.9)	36.1 (33.3–38.9)	5.6 (4.4–7.1)	17.2 (15.1–19.5)
25–34	2,631 (53.9)	38.1 (36.3–40.1)	8.1 (7.1–9.2)	22.1 (20.6–23.8)
35–49	1,130 (23.1)	34.8 (32.1–37.6)	6.7 (5.4–8.3)	22.3 (20.0–24.8)
**Educational level**				
No education	2,616 (53.6)	41.7 (39.8–43.6)	9.2 (8.2–10.4)	26.1 (24.5–27.8)
Primary	1,725 (35.4)	35.4 (33.1–37.6)	4.9 (4.0–6.1)	17.3 (15.6–19.1)
Secondary	359 (7.3)	18.9 (15.2–23.3)	5.7 (3.7–8.6)	9.3 (6.7–12.8)
Higher	179 (3.7)	17.0 (12.2–23.2)	1.3 (0.3–4.5)	6.3 (3.5–10.9)
**ANC visit**				
No ANC	872 (24.6)	33.4 (30.4–36.6)	9.7 (7.9–11.8)	21.8 (19.2–24.7)
1–3	1,117 (31.6)	35.9 (33.2–38.8)	8.1 (6.7–9.9)	22.9 (20.5–25.4)
4+	1,550 (43.8)	34.5 (32.2–36.9)	4.4 (3.5–5.6)	16.3 (14.5–18.2)
**Maternal postnatal visit**				
Yes	506 (13.9)	35.4 (31.4–39.7)	4.5 (2.9–6.7)	18.7 (15.5–22.3)
No	3,141 (86.1)	34.7 (32.9–36.4)	7.3 (6.4–8.3)	19.9 (18.5–21.3)
**Place of delivery**				
Home	2,487 (51.5)	39.6 (37.7–41.5)	8.5 (7.5–9.7)	23.8 (22.2–25.5)
Health facility	2,340 (48.5)	34.5 (32.6–36.4)	5.7 (4.8–6.7)	18.2 (16.7–19.8)
**Household-related factors**				
**Wealth index**				
Poorest	1,122 (22.9)	42.7 (39.8–45.6)	11.5 (9.8–13.5)	28.8 (26.3–31.5)
Poorer	1,076 (22.1)	38.9 (36.0–41.8)	7.4 (5.9–9.1)	23.3 (20.9–25.9)
Middle	916 (18.8)	42.4 (39.2–45.5)	5.1 (3.8–6.7)	22.3 (19.7–25.1)
Richer	860 (17.6)	36.1 (32.9–39.3)	6.4 (4.9–8.2)	16.6 (14.3–19.3)
Richest	904 (18.5)	22.5 (19.9–25.4)	4.2 (3.1–5.7)	11.5 (9.6–13.7)
**Floor**				
Natural/Earth	4,041 (83.3)	39.9 (38.5–41.4)	7.8 (6.9–8.6)	23.3 (22.0–24.6)
Improved	808 (16.7)	21.7 (18.9–24.6)	4.4 (3.2–6.1)	10.3 (8.4–12.6)
**Household crowded** ^**#**^				
Yes	4,564 (86.8)	37.8 (36.4–39.3)	7.4 (6.6–8.2)	22.4 (21.2–23.6)
No	694 (13.2)	29.5 (26.3–33.0)	4.3 (3.1–6.1)	13.5 (11.1–16.2)
**Toilet facility**				
Improved	730 (15.1)	28.6 (25.4–32.0)	7.2 (5.5–9.2)	18.6 (16.0–21.6)
Unimproved	1,545 (32.0)	36.6 (34.8–38.5)	5.4 (4.6–6.4)	18.9 (17.5–20.5)
Open defecation	2,550 (52.9)	41.7 (39.3–44.2)	10.2 (8.8–11.8)	26.2 (24.0–28.4)
**Source of drinking water**				
Improved	3,168 (65.3)	36.5 (34.9–38.2)	7.1 (6.3–8.1)	20.7 (19.4–22.2)
Unimproved	918 (18.9)	38.9 (35.9–42.1)	6.1 (4.8–7.9)	20.7 (18.2–23.4)
Surface water	766 (15.8)	36.2 (32.8–39.6)	8.9 (7.1–11.1)	23.1 (20.3–26.2)
**Time to water source**				
On premises	674 (13.9)	27.4 (24.1–30.8)	4.7 (3.3–6.5)	13.2 (10.8–15.9)
< 30 min	1,923 (39.7)	38.2 (36.0–40.4)	7.6 (6.5–8.9)	23.5 (21.6–25.4)
> = 30 min	2,248 (46.4)	38.5 (36.6–40.6)	7.5 (6.5–8.7)	21.4 (19.7–23.1)
**Community-level factor**				
**Residence**				
Urban	1,218 (24.9)	25.6 (23.2–28.1)	5.71 (4.5–7.1)	14.2 (12.3–16. 2)
Rural	3,660 (75.1)	40.7 (39.1–42.3)	7.7 (6.8–8.6)	23.3 (22.0–24.7)
**Region**				
Agrarian	4,294 (88.0)	38.1 (36.6–39.5)	6.1 (5.4–6.8)	20.6 (19.4–21.8)
Pastoralist	407 (8.4)	32.9 (28.5–37.6)	19.9 (16.4–24.1)	31.9 (27.6–36.5)
Metropolis	176 (3.6)	17.2 (12.3–23.4)	3.0 (1.3–6.8)	7.3 (4.3–12.1)

#: If three or more people living in the same room considered crowded.

### Prevalence of childhood undernutrition

**Fig 2:**
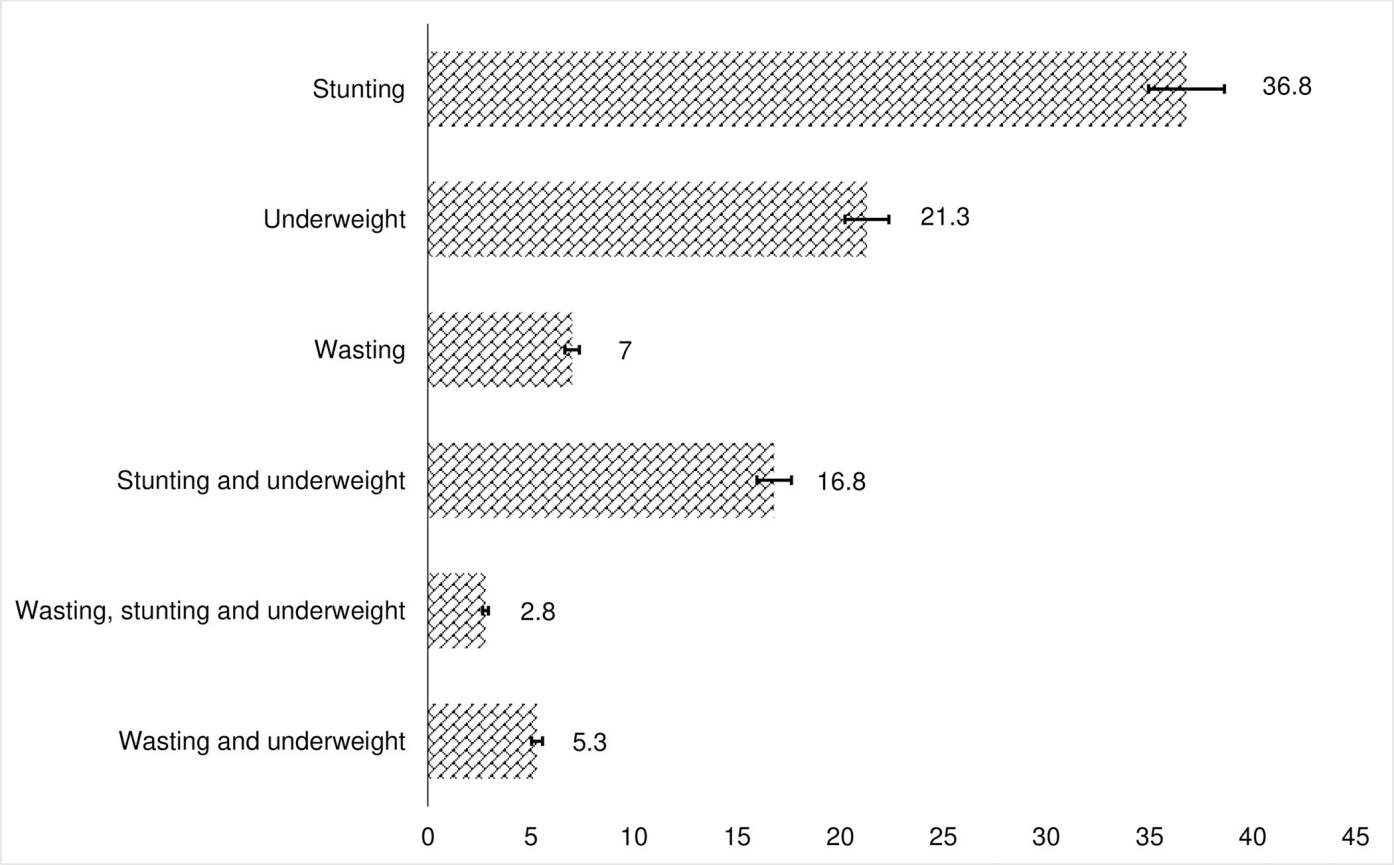
Combined/Aggregate CIAF in children under 5 years in Ethiopia.

Regarding children’s undernutrition status, about 36.8% (95%C: 35.5–38.1), 21.3% (95%CI: 20.2–22.4), and 7.0% (95%CI: 6.4–7.7) of the children were stunted, underweight and wasted, respectively (Table 2). Fig 2 illustrates the combined/aggregate CIAF in children under 5 years in Ethiopia. As shown in Fig 2, the prevalence of co-existing stunting and underweight among children aged 0–59 months was 16.8 (95%CI: 15.8–17.9). Table 3 shows the prevalence of severe undernutrition among the children aged 0–59 months in Ethiopia. The prevalence of severe stunting, severe wasting, and severe underweight were 8.1%, 0.5%, and 0.7%, respectively. The overall prevalence of undernutrition using the CIAF and CISAF among the children under 5 years old was 42.2% (95%CI: 40.9–43.5) and 13.9% (95%CI: 12.9–14.8), respectively.

**Table 2:** Distribution of participants as per CIAF categories, EMDHS-2019, (n = 5,259).

Group	Categories of undernutrition	Wasting	Stunting	Underweight	n	Prevalence (95%CI)
A	No failure	No	No	No	3,041	57.8 (56.5–59.2)
B	Wasting	Yes	No	No	379	7.0 (6.4–7.7)
C	Wasting and underweight	Yes	No	Yes	282	5.3 (4.7–5.9)
D	Wasting, stunting and underweight	Yes	No	Yes	146	2.8 (2.4–3.2)
E	Stunting and underweight				889	16.8 (15.8–17.9)
F	Stunting	No	Yes	No	1,941	36.8 (35.5–38.1)
Y	Underweight	No	No	Yes	1,135	21.3 (20.2–22.4)

**Table 3:** Prevalence of severe undernutrition among the children aged 0–59 months as per CISAF categories, EMDHS-2019, (n = 5,259).

Group	Categories of severe undernutrition	Severe wasting	Severe stunting	Severe underweight	n	Prevalence (95%CI)
A	No severe failure	No	No	No	4,519	86.1 (85.1–87.1)
B	Severe wasting only	Yes	No	No	26	0.5 (0.3–0.7)
C	Severe wasting and severe underweight	Yes	No	Yes	15	0.3 (0.1–0.5)
D	Severe wasting, severe stunting and severe underweight	Yes	Yes	Yes	16	0.3 (0.1–0.5)
E	Severe stunting and severe underweight	No	Yes	Yes	209	3.9 (3.5–4.5)
F	Severe stunting only	No	Yes	No	424	8.1 (7.3–8.8)
Y	Severe underweight only	No	No	Yes	39	0.7 (0.5–1.0)

### Factors associated with undernutrition using CIAF

Several factors were found to be significantly associated with both CIAF and CISAF failure among children under the age of five. These factors include being a male child, falling within the 12–59 months age group, residing in households with multiple under-five children, having a mother with primary or higher education, and belonging to a family with a poorer wealth index. Additionally, CIAF demonstrated a significant association with housing status and place of residence.

In the bivariate multilevel mixed-effect negative binomial regression analysis, several individual and community-level factors were associated with CIAF (Table 4).

**Table 4:** Bivariate multilevel mixed-effects negative binomial regression analysis of factors associated with CIAF, EMDHS 2019 (n = 5,259).

Characteristics	Anthropometric failure using the CIAF, % (95%CI)	Unadjusted IRR (95%CI)	p-value
**Child-related**			
**Sex**			
Male	45.9 (43.9–47.9)	1.17 (1.08–1.25)	p<0.001
Female	38.6 (36.7–40.6)	Ref.	
**Age (months)**			
<6	25.7 (22.1–29.7)	Ref.	
6–11	33.5 (29.4–37.9)	1.22 (1.01–1.47)	0.041
12–24	38.6 (35.7–41.4)	1.71 (1.46–2.00)	p<0.001
25–35	50.6 (47.2–53.9)	2.19 (1.87–2.56)	p<0.001
36–59	47.4 (45.2–49.6)	1.91 (1.65–2.22)	p<0.001
**Number of under-five children**			
1	38.3 (36.1–40.4)	Ref.	
2+	45.0 (43.2–46.8)	1.08 (1.01–1.17)	0.043
**Birth interval**			
< 24 months	48.9 (45.4–52.4)	Ref.	
≥ 24 months	41.9 (40.1–43.7)	0.84 (0.77–0.92)	p<0.001
**Vitamin A in last 6 months (n = 2,880)**			
Yes	37.2 (34.8–39.6)	Ref.	
No	41.1 (38.4–43.8)	1.15 (1.03–1.28)	0.011
**Currently breastfeeding**			
Yes	37.8 (35.7–39.8)	0.82 (0.76–0.89)	p<0.001
No	46.2 (44.3–48.1)	Ref.	
**Minimum meal frequency**			
Yes	37.3 (34.7–39.8)	0.85 (0.78–0.94)	0.001
No	43.9 (42.3–45.5)	Ref.	
**Minimum dietary diversity**			
Yes	28.4 (22.9–34.6)	0.64 (0.50–0.81)	p<0.001
No	43.0 (41.6–44.4)	Ref.	
**Maternal factors**			
**Age (years)**			
15–24	40.4 (37.6–43.3)	Ref.	
25–34	43.9 (42.1–45.8)	1.05 (0.96–1.15)	0.263
35–49	40.4 (37.6–43.3)	1.02 (0.91–1.14)	0.735
**Educational level**			
No education	48.1 (46.2–50.1)	Ref.	
Primary	39.7 (37.4–41.9)	0.84 (0.77–0.91)	p<0.001
Secondary	23.6 (19.5–28.3)	0.52 (0.43–0.62)	p<0.001
Higher	20.4 (15.1–26.9)	0.40 (0.31–0.52)	p<0.001
**ANC visit**			
No ANC	40.4 (37.1–43.6)	Ref.	
1–3	41.9 (39.1–44.9)	0.89 (0.79–1.04)	0.059
4+	38.5 (36.1–40.9)	0.78 (0.68–0.88)	p<0.001
**Maternal postnatal visit**			
Yes	506 (13.9)	Ref.	
No	3,141 (86.1)	1.02 (0.89–1.17)	0.737
**Place of delivery**			
Home	45.9 (43.9–47.9)	Ref.	
Health facility	38.8 (36.9–40.8)	0.83 (0.76–0.91)	p<0.001
**Household-level factors**			
**Wealth index**			
Poorest	50.8 (47.8–53.7)	2.14 (1.86–2.46)	p<0.001
Poorer	44.8 (41.9–47.8)	1.93 (1.66–2.23)	p<0.001
Middle	46.4 (43.2–49.7)	1.82 (1.56–2.12)	p<0.001
Richer	40.5 (37.3–43.8)	1.55 (1.32–1.81)	p<0.001
Richest	26.4 (23.7–29.4)	Ref.	
**Floor**			
Natural/Earth	45.8 (44.3–47.3)	1.76 (1.56–1.99)	p<0.001
Improved	25.4 (22.5–28.5)	Ref.	
**Household crowded** ^**#**^			
Yes	43.6 (42.1–45.0)	1.32 (1.16–1.51)	p<0.001
No	32.7 (29.4–36.3)	Ref.	
**Toilet facility**			
Improved	31.2 (28.5–34.2)	Ref.	
Unimproved	45.5 (44.1–47.1)	1.36 (1.21–1.53)	p<0.001
**Source of drinking water**			
Improved	39.6 (37.9–41.3)	Ref.	
Unimproved	48.3 (45.9–50.6)	1.17 (1.07–1.29)	0.001
**Time to water source**			
On-premises	30.5 (27.1–34.1)	Ref.	
< 30 min	44.0 (41.8–46.3)	1.70 (1.47–1.97)	p<0.001
> = 30 min	44.4 (42.3–46.4)	1.64 (1.42–1.89)	p<0.001
**Community-level factors**			
**Residence**			
Urban	30.4 (27.9–33.1)	Ref.	
Rural	46.3 (44.7–47.9)	1.78 (1.54–2.06)	p<0.001
**Region**			
Agrarian	42.7 (41.2–44.2)	Ref.	
Pastoralist	48.5 (43.7–53.4)	1.30 (1.12–1.51)	0.001
Metropolis	19.6 (14.3–26.1)	0.61 (0.52–0.72)	p<0.001

Table 5 presents the multivariable regression analysis results. The expected log counts of children suffering from a composite index of anthropometric failure (CIAF) were higher for male children (aIRR = 1.27, 95% CI = 1.13–1.42) than for females. Children in the age group of 12–24 and 24–59 months had 2.01 (aIRR = 2.01, 95%CI: 1.63–2.48), and 2.36 (aIRR = 2.36, 95%CI: 1.91–2.92) times higher incidence-rate ratios of CIAF compared with those < 6 months of age, respectively. The expected log counts of children suffering from a CIAF were higher for children from households living with two and more under-five children than their counterparts (aIRR = 1.16, 95%CI: 1.01–1.33). The incidence-rate ratios of CIAF was significantly lower for children living with mothers having better education (i.e., secondary education (aIRR = 0.58, 95%CI (0.42–0.79) and higher education (aIRR = 0.36, 95%CI: 0.21–0.61)). The incidence-rate ratios of children suffering from a composite index of anthropometric failure were higher for children from poorer households (aIRR = 1.48, 95% CI = 1.02–2.15) compared with children from relatively richest households. The incidence-rate ratios of CIAF was higher for children from households living on earthen floors compared to those living in improved floor households (aIRR = 1.37, 95%CI: 1.03–1.82). The incidence-rate ratios of children suffering from a composite index of anthropometric failure were decreased by about 26% for children from rural areas compared to that of urban residence (aIRR = 0.74, 95% CI: 0.59–0.93).

**Table 5:** Multivariable multilevel mixed-effects negative binomial regression analysis of factors associated with CIAF, EMDHS 2019 (n = 5,259).

Characteristics	Model 1	Model 2	Model 3	Model 4
	Null Model	aIRR (95%CI)	aIRR (95%CI)	aIRR (95%CI)
**Child-related**				
**Sex**				
Male		1.28 (1.14–1.43)**		1.27 (1.13–1.42)**
Female		Ref.		Ref.
**Age (months)**				
<6		Ref.		Ref.
6–11		1.28 (1.01–1.62)*		1.26 (0.99–1.60)
12–24		2.02 (1.64–2.50)**		2.01 (1.63–2.48)**
25–59		2.37 (1.91–2.94)**		2.36 (1.91–2.92)**
**Number of under-five children**				
1		Ref.		Ref.
2+		1.17 (1.02–1.34)*		1.16 (1.01–1.33)*
**Birth interval**				
< 24 months		Ref.		Ref.
≥ 24 months		0.92 (0.80–1.07)		0.93 (0.81–1.08)
**Vitamin A in last 6 months (n = 2,880)**				
Yes		Ref.		Ref.
No		1.08 (0.95–1.23)		1.08 (0.95–1.23)
**Currently breastfeeding**				
Yes		1.05 (0.91–1.20)		1.05 (0.91–1.20)
No		Ref.		Ref.
**Minimum meal frequency**				
Yes		0.91 (0.77–1.06)		0.91 (0.78–1.07)
No		Ref.		Ref.
**Minimum dietary diversity**				
Yes		0.83 (0.61–1.13)		0.83 (0.61–1.13)
No		Ref.		Ref.
**Maternal factors**				
**Age (years)**				
15–24		Ref.		Ref.
25–34		1.07 (0.91–1.27)		1.08 (0.91–1.27)
35–49		1.02 (0.83–1.24)		1.02 (0.83–1.24)
**Educational level**				
No education		Ref.		Ref.
Primary		0.92 (0.80–1.06)		0.92 (0.80–1.06)
Secondary		0.58 (0.42–0.80)		0.58 (0.42–0.79)*
Higher		0.37 (0.21–0.62)		0.36 (0.21–0.61)**
**ANC visit**				
No ANC		Ref.		Ref.
1–3		1.01 (0.87–1.18)		1.03 (0.88–1.20)
4+		1.07 (0.91–1.27)		1.09 (0.92–1.29)
**Place of delivery**				
Home		Ref.		Ref.
Health facility		0.97 (0.84–1.12)		0.97 (0.84–1.12)
**Household-level factors**				
**Wealth index**				
Poorest		1.39 (0.98–1.98)		1.42 (0.96–2.10)
Poorer		1.32 (0.94–1.87)		1.48 (1.02–2.15)*
Middle		1.18 (0.83–1.66)		1.34 (0.93–1.95)
Richer		1.17 (0.84–1.61)		1.29 (0.92–1.80)
Richest		Ref.		Ref.
**Floor**				
Natural/Earth		1.39 (1.05–1.83)*		1.37 (1.03–1.82)*
Improved		Ref.		Ref.
**Household crowded** ^**#**^				
Yes		1.19 (0.95–1.48)		1.19 (0.96–1.49)
No		Ref.		Ref.
**Toilet facility**				
Improved		Ref.		Ref.
Unimproved		0.85 (0.69–1.04)		0.86 (0.71–1.05)
**Source of drinking water**				
Improved		Ref.		Ref.
Unimproved		0.99 (0.86–1.15)		1.01 (0.87–1.15)
**Time to water source**				
On-premises		Ref.		Ref.
< 30 min		1.08 (0.83–1.39)		1.14 (0.88–1.48)
≥ 30 min		0.98 (0.76–1.28)		1.03 (0.79–1.34)
**Community-level factors**				
**Residence**				
Urban			Ref.	Ref.
Rural			1.54 (1.32–1.79)**	0.74 (0.59–0.93)*
**Region**				
Agrarian			Ref.	Ref.
Pastoralist			1.32 (1.14–1.53)*	1.09 (0.90–1.32)
Metropolis			0.75 (0.63–0.89)*	0.89 (0.71–1.11)
**Random effect**				
Cluster level variance (SE)	0.191 (0.029)**	0.064 (0.025)**	0.128 (0.0212)**	0.055 (0.025)*
ICC (%)	5.49	1.91	3.73	1.65
PCV (%)	Ref.	65.2	32.1	69.9
MIRR	1.66	1.34	1.51	1.31
**Model comparison**				
Log-likelihood (LL)	-5560.37	-2096.23	-5513.70	-2091.37
Deviance	11,120.74	4,192.46	11,027.40	4,182.74
AIC	11126.76	4258.479	11039.41	4254.75
BIC	11146.33	4443.979	11078.57	4457.113

**p<0.001

*p<0.05

ICC: Intra-class Correction Coefficient; MIRR = median incident rate ratio; PCV: Proportional Change in Variance; AIC: Akaike’s Information Criterion; BIC: Bayesian Information Criteria; LL: Log-likelihood; Model 1 is the null model, a baseline model without any independent variable; Model 2 is adjusted for individual-level factors; Model 3 is adjusted for community-level factors; Model 4 is the full model adjusted for both individual and community-level factors.

### Factors associated with undernutrition using CISAF

Tables 6 and 7 presents the bivariable and multivariable multilevel mixed-effect negative binomial regression analysis results of CISAF, respectively.

**Table 6:** Bivariate multilevel mixed-effects negative binomial regression analysis of factors associated with CISAF, EMDHS 2019 (n = 5,259).

Characteristics	Anthropometric failure using the CISAF, % (95%CI)	Unadjusted IRR (95%CI)	p-value
**Child-related**			
**Sex**			
Male	16.6 (15.2–18.0)	1.31 (1.13–1.52)	p<0.001
Female	11.1 (9.9–12.4)	Ref.	
**Age (months)**			
<6	5.6 (3.9–8.0)	Ref.	
6–11	11.7 (9.1–14.9)	1.45 (0.97–2.15)	0.066
12–24	11.1 (9.4–13.1)	2.00 (1.44–2.79)	p<0.001
25–35	18.2 (15.7–20.9)	2.97 (2.14–4.13)	p<0.001
36–59	15.9 (14.4–17.6)	2.32 (1.70–3.17)	p<0.001
**Number of under-five children**			
1	11.3 (9.9–12.8)	Ref.	
2+	15.3 (14.1–16.7)	1.11 (0.95–1.31)	0.189
**Birth interval**			
< 24 months	19.4 (16.4–21.9)	Ref.	
≥ 24 months	13.8 (12.6–15.0)	0.77 (0.64–0.93)	0.007
**Vitamin A in last 6 months**			
Yes	11.8 (10.3–13.5)	Ref.	
No	12.3 (10.6–14.2)	1.08 (0.86–1.33)	0.494
**Currently breastfeeding**			
Yes	13.6 (12.4–14.8)	0.96 (0.81–1.14)	0.643
No	13.5 (11.7–15.6)	Ref.	
**Minimum meal frequency**			
Yes	11.3 (9.7–13.0)	0.70 (0.58–0.85)	p<0.001
No	14.8 (13.7–15.9)	Ref.	
**Minimum dietary diversity**			
Yes	8.8 (5.7–13.3)	0.51 (0.31–0.86)	0.011
No	13.9 (13.0–15.1)	Ref.	
**Maternal factors**			
**Age (years)**			
15–24	11.0 (9.3–13.0)	Ref.	
25–34	14.9 (13.7–16.4)	1.14 (0.95–1.38)	0.151
35–49	13.5 (11.7–15.6)	1.12 (0.89–1.41)	0.314
**Educational level**			
No education	18.0 (16.6–19.6)	Ref.	
Primary	10.4 (9.0–11.9)	0.63 (0.53–0.75)	p<0.001
Secondary	4.4 (2.7–7.1)	0.26 (0.17–0.40)	p<0.001
Higher	1.4 (0.4–4.7)	0.09 (0.04–0.22)	p<0.001
**ANC visit**			
No ANC	14.4 (12.2–16.9)	Ref.	
1–3	14.5 (12.6–16.7)	0.84 (0.67–1.06)	0.157
4+	10.6 (9.1–12.2)	0.59 (0.47–0.76)	p<0.001
**Maternal postnatal visit**			
Yes	506 (13.9)	Ref.	
No	3,141 (86.1)	1.25 (0.94–1.67)	0.121
**Place of delivery**			
Home	16.9 (15.5–18.5)	Ref.	
Health facility	10.4 (9.3–11.7)	0.62 (0.53–0.74)	p<0.001
**Household-level factors**			
**Wealth index**			
Poorest	19.7 (17.5–22.1)	3.93 (2.95–5.25)	p<0.001
Poorer	16.0 (13.9–18.3)	3.35 (2.47–4.56)	p<0.001
Middle	14.3 (12.1–16.7)	2.75 (1.99–3.82)	p<0.001
Richer	11.9 (9.9–14.2)	2.19 (1.56–3.06)	p<0.001
Richest	4.7 (3.5–6.4)	Ref.	
**Floor**			
Natural/Earth	15.6 (14.5–16.7)	2.80 (2.16–3.62)	p<0.001
Improved	4.8 (3.5–6.5)	Ref.	
**Household crowded** ^**#**^			
Yes	14.6 (13.6–15.7)	1.46 (1.11–1.92)	0.007
No	9.2 (7.2–11.6)	Ref.	
**Toilet facility**			
Improved	10.4 (8.4–12.8)	Ref.	
Unimproved	14.4 (13.4–15.5)	1.78 (1.39–2.26)	p<0.001
**Source of drinking water**			
Improved	13.1 (12.0–14.4)	Ref.	
Unimproved	15.0 (13.4–16.8)	1.40 (1.16–1.69)	p<0.001
**Time to water source**			
On-premises	6.6 (5.0–8.8)	Ref.	
< 30 min	16.0 (14.5–17.7)	2.63 (1.95–3.56)	p<0.001
> = 30 min	13.9 (12.5–15.4)	2.55 (1.89–3.44)	p<0.001
**Community-level factors**			
**Residence**			
Urban	6.8 (5.5–8.4)	Ref.	
Rural	16.0 (14.8–17.2)	2.71 (2.04–3.61)	p<0.001
**Region**			
Agrarian	13.5 (12.5–14.6)	Ref.	
Pastoralist	19.3 (15.7–23.3)	1.68 (1.29–2.18)	p<0.001
Metropolis	5.3 (2.8–9.8)	0.52 (0.38–0.71)	p<0.001

**Table 7:** Multivariable multilevel mixed-effects negative binomial regression analysis of factors associated with CISAF, EMDHS 2019 (n = 5,259).

Characteristics	Model 1	Model 2	Model 3	Model 4
	Null Model	aIRR (95%CI)	aIRR (95%CI)	aIRR (95%CI)
**Child-related**				
**Sex**				
Male		1.48 (1.22–1.81)**		1.47 (1.21–1.79)**
Female		Ref.		Ref.
**Age (months)**				
<6		Ref.		Ref.
6–11		2.29 (1.39–3.76)*		2.30 (1.40–3.78)*
12–24		3.70 (2.37–5.79)**		3.76 (2.40–5.88)**
25–59		4.68 (3.05–7.17)**		4.23 (2.79–6.39)**
**Number of under-five children**				
1		Ref.		Ref.
2+		1.21 (0.95–1.54)		1.27 (1.01–1.59)*
**Birth interval**				
< 24 months		Ref.		Ref.
≥ 24 months		0.92 (0.72–1.18)		0.97 (0.76–1.25)
**Minimum meal frequency**				
Yes		0.75 (0.55–1.02)		0.74 (0.54–1.01)
No		Ref.		Ref.
**Minimum dietary diversity**				
Yes		1.14 (0.61–2.11)		1.19 (0.64–2.21)
No		Ref.		Ref.
**Maternal factors**				
**Age (years)**				
15–24		Ref.		Ref.
25–34		1.03 (0.76–1.38)		1.07 (0.79–1.44)
35–49		0.96 (0.68–1.35)		1.01 (0.72–1.42)
**Educational level**				
No education		Ref.		Ref.
Primary		0.75 (0.58–0.96)*		0.79 (0.61–1.01)
Secondary		0.41 (0.22–0.75)*		0.42 (0.23–0.77)*
Higher		0.06 (0.01–0.46)*		0.06 (0.01–0.48)*
**ANC visit**				
No ANC		Ref.		Ref.
1–3		1.10 (0.85–1.42)		1.15 (0.89–1.50)
4+		1.11 (0.83–1.48)		1.18 (0.89–1.58)
**Maternal postnatal visit**				
Yes		Ref.		Ref.
No		1.13 (0.83–1.55)		1.11 (0.82–1.51)
**Place of delivery**				
Home		Ref.		Ref.
Health facility		0.92 (0.72–1.17)		0.93 (0.73–1.19)
**Household-level factors**				
**Wealth index**				
Poorest		1.85 (1.01–3.42)*		1.79 (0.93–3.49)
Poorer		1.76 (0.94–3.21)		1.93 (1.02–3.67)*
Middle		1.56 (0.83–2.84)		1.75 (0.92–3.31)
Richer		1.29 (0.84–2.27)		1.38 (0.76–2.50)
Richest		Ref.		Ref.
**Floor**				
Natural/Earth		1.13 (0.71–1.80)		1.25 (0.77–2.01)
Improved		Ref.		Ref.
**Household crowded** ^**#**^				
Yes		0.98 (0.68–1.39)		0.98 (0.69–1.41)
No		Ref.		Ref.
**Toilet facility**				
Improved		Ref.		Ref.
Unimproved		1.12 (0.77–1.62)		1.21 (0.83–1.75)
**Source of drinking water**				
Improved		Ref.		Ref.
Unimproved		1.09 (0.87–1.38)		1.09 (0.86–1.38)
**Time to water source**				
On-premises		Ref.		Ref.
< 30 min		1.35 (0.85–2.14)		1.55 (0.97–2.47)
≥ 30 min		1.29 (0.81–2.06)		1.38 (0.86–2.20)
**Community-level factors**				
**Residence**				
Urban			Ref.	Ref.
Rural			2.35 (1.75–3.16)**	0.83 (0.55–1.26)
**Region**				
Agrarian			Ref.	Ref.
Pastoralist			1.73 (1.35–2.23)**	1.60 (1.16–2.20)
Metropolis			0.75 (0.55–1.03)	1.30 (0.89–1.91)
**Random effect**				
Cluster level variance (SE)	0.434 (0.084)**	0.175 (0.073)*	0.305 (0.066)**	0.155 (0.070)*
ICC (%)	11.67	5.05	8.48	4.49
PCV (%)	Ref.	59.67	29.7	64.28
MIRR	2.14	1.62	1.89	1.58
**Model comparison**				
Log-likelihood (LL)	-2745.84	-1444.91	-2706.83	-1441.68
Deviance	5,491.68	2,889.83	5,413.67	2,883.36
AIC	5497.688	2949.83	5425.67	2947.36
BIC	5517.266	3127.58	5464.83	3136.95

**p<0.001

*p<0.05

ICC: Intra-class Correction Coefficient; MIRR = median incident rate ratio; PCV: Proportional Change in Variance; AIC: Akaike’s Information Criterion; BIC: Bayesian Information Criteria; LL: Log-likelihood; Model 1 is the null model, a baseline model without any independent variable; Model 2 is adjusted for individual-level factors; Model 3 is adjusted for community-level factors; Model 4 is the full model adjusted for both individual and community-level factors.

The key factors positively associated with CISAF among children aged 0–59 months were being male children (aIRR = 1.47, 95% CI = 1.21–1.79), age group 6–11 months (aIRR = 2.30, 95%CI: 1.40–3.78), age group 12–24 months (aIRR = 3.76, 95%CI: 2.40–5.88), age group 25–59 months (aIRR = 4.23, 95%CI: 2.79–6.39), children from households living with two and more under-five children (aIRR = 1.27, 95%CI:1.01–1.59), and children from poorer households (aIRR = 1.93, 95% CI = 1.02–3.67). In contrast, children living with mothers who had completed secondary education (aIRR = 0.42, 95%CI (0.23–0.77)) and higher education (aIRR = 0.06, 95%CI: 0.01–0.48) had significantly reduced incidence-rate ratios of CISAF than children whose mothers had not completed any education.

## Discussion

Undernutrition at early ages is linked to lifelong consequences, such as reduced cognitive skills, reduced earnings in adulthood, and chronic health conditions. Ethiopia has committed to addressing child undernutrition, and ending hunger as a foundational to achieving the set Sustainable Development Goals. A single anthropometric index, such as stunting, wasting, or underweight, does not provide a complete depiction of childhood malnutrition in children under the age of five. The composite index of anthropometric failure (CIAF) and the composite index of severe anthropometric failure (CISAF), an aggregated single anthropometric measure, provides an overall estimate of undernourished children [24] and portrays a better state of undernutrition in a population or a nation. This study intended to identify the determinants of the CIAF and CISAF of children under five years old in Ethiopia based on EMDHS 2019 data. The multilevel negative binomial regression analysis revealed that the common predictors significant associated with both CIAF and CISAF includes sex of the child, age of the child, number of under-five children, mothers’ education level, and household wealth status.

In this study, CIAF identified undernutrition in 42.2% of the children overall, while conventional anthropometric indices showed 36.8% stunting, 7.0% wasting and 21.3% underweight. The prevalence of CIAF observed in our study was marginally lower compared to the figures reported in other developing nations such as Bangladesh, Pakistan, India and Indonesia [27–29,47]. The overall prevalence of severe undernutrition measured by CISAF among children under 5 was 13.9%. This finding aligns with a study from Bangladesh, which reported a prevalence of CISAF among children under five was 11.0% [31].

The sex of the child is an important demographic variable that affects the nutritional status of children. In the current study, the incidence-rate ratios of children suffering from a composite index of anthropometric failure were higher for male children than for females. Similarly, the expected log counts of children suffering from a CISAF were higher for male children than for females. The observed gender differences in anthropometric failure have been reported in earlier studies and is consistent with previous findings from Nigeria [48], Rwanda [49], and Senegal [50]. Similarly, studies from Asian countries, like India [51], Pakistan [52], and Indonesia [53] also reported related findings. A systematic review and meta-analysis by Thurstans et al also found that boys had a higher risk of stunting, wasting, and being underweight than girls [54]. The possible causal direction of undernutrition and gender and the possible mechanism that explains this interaction in children aged under five years remain unclear and warrant further studies. Possible explanations, however, have been linked to variations in immune system development [55] and biological factors [54]. Still, additional research seems to be necessary to understand why male children are more likely to be undernourished when compared with their female counterparts.

In this study, older children were found to be at a higher risk of developing CIAF and CISAF compared to those aged less than 6 months. This could be attributed to several factors. Firstly, younger children are more likely to exclusively breastfeed, which provides essential nutrients and boosts immunity, reducing the risk of undernutrition and associated conditions [56]. Additionally, as children grow older, they are introduced to complementary foods, but these may not always be adequately nutritious, leading to an increased risk of undernutrition. Moreover, older children are more susceptible to infections and diseases due to their increased exposure to the environment and decreased immunity compared to infants under six months. These factors collectively contribute to the higher risk of anthropometric failure observed in older children as compared to their younger counterparts [57–59].

In this current study, we found that children aged 12–24 months and 24–59 months were more likely to suffer CIAF and CISAF compared to their counterparts aged less than 12 months, which is consistent with past studies in different settings [60,61]. Undernutrition in older children can be explained by the following: a lack of adequate and balanced food intake to meet the metabolic demand for childhood growth as they age and older children’s frequent interactions with their unhygienic surroundings, which may increase the risks of exposure to childhood infectious diseases such as diarrheal diseases, parasite infections (hookworms, roundworms etc.), and other acute illnesses, all of which increase the risk of childhood undernutrition [62].

Our study revealed that there was a significant decline in the expected rate ratio of multiple nutritional failures for children with higher maternal education than and no education. This finding is not unexpected, given that maternal higher education had a protective effect on CIAF [63]. This finding is also consistent with previous studies that found low maternal education was strongly associated with poor nutritional outcomes in children [64]. These findings were also matched with the previous study in Bangladesh, which reported that CISAF was more dominant among the children of uneducated parents [31].

The incidence-rate ratios of children suffering from a composite index of anthropometric failure and severe anthropometric failure were higher for children from poorer households than richest households. Our findings are consistent with other studies showing strong positive associations between childhood undernutrition and household wealth status [31,65]. Children from relatively wealthy households were shown to be less likely to be undernourished, since wealth status serves as a substitute for greater socioeconomic position, improving mothers’ ability to afford the cost of healthy food and ensuring household food security. Our study also emphasized that under-five children from households living with two and more under-five children and living in earthen floor households are at a higher risk of suffering from CIAF. These findings are not surprising given that education, housing and poverty are important social determinants of health (SDoH), conditions in which people are born, grow, work, live, and age [66]. Synonymous to the SDoH framework, the causes of undernutrition are multifactorial and interlinked with the environment under which these children live and grow. Importantly, this study found that children from households with earthen flooring had an increased likelihood of developing CIAF. Our findings are consistent with the earlier cross-sectional study’s findings that improved housing was associated with lower odds of stunting. But the composite index for measuring undernutrition, that is CIAF was not applied in this study [67]. Our findings support the idea that nutrition interventions alone are unlikely to improve childhood nutritional problems. On the other hand, current evidence clearly shows that children’s growth and development are influenced by their housing environment [68], supporting the current study findings and the social determinant of health framework [66]. The other finding of this study is that the incidence-rate ratios of children suffering from the composite index of anthropometric failure were lower for children from rural areas than urban dwellers, which was somewhat surprising, and contrary to other studies [69].

This study’s strength is using a nationwide population-based dataset that provides a large sample size and statistical power to identify the factors associated with CIAF in Ethiopia. However, there are some limitations; first, as the current study was cross-sectional in design, the causal direction between risk factors and CIAF cannot be ascertained. Second, this study employed secondary data, it did not account for factors that could affect the occurrence of CIAF, such as food security and health-related factors. Third, the recall bias might have occurred because of the self-reported nature of some of the variables. Fourth, in contrast to other studies, this study may be limited because our analysis did not take into account some factors such as the mother’s BMI and the child’s size at birth, which were not captured in EMDHS. In spite of this limitations, we used EMDHS over the standard Ethiopian Demographic and Health Survey (EDHS), for several reason: one of the primary reasons for using the EMDHS is its timeliness and relevance. The EMDHS provides more recent data compared to the standard EDHS, which was conducted in 2016. Given that demographic and health indicators can change over time, having access to more current data is crucial for making informed decisions and policies. Policymakers may prioritize utilizing the most recent data available to inform their decision-making processes.

## Conclusions

Our study highlighted the key drivers of CIAF and CISAF in children aged less than five years in Ethiopia; including male sex, older age, living in households having two and more under-five children, children from poorer households, and living in poor housing condition. The expected log counts of children suffering from CIAF and CISAF were lower among children born to mothers with higher education. Given the high scale of malnutrition in Ethiopia, there is an urgent need to accelerate efforts to address the multiple anthropometric failures through a holistic approach such as double-duty actions, which is an efficient way to tackle these co-existing forms of malnutrition simultaneously through nutrition-specific and nutrition-sensitive interventions. Finally, we recommend using single composite indicators such as CIAF and CISAF to determine childhood undernutrition in order to develop effective interventions in Ethiopia, where childhood undernutrition is endemic. The use of a composite index of anthropometric failure can also contribute to achieving the SDGs in Ethiopia in several ways. Primarily, it can help identify populations most affected by undernutrition and inform targeted interventions to accelerate progress towards the SDGs. Second, the CIAF can be used to monitor progress towards the SDGs related to nutrition, such as SDG 2 (zero hunger).

## Supporting information

S1 Table Classification of composite index of anthropometric failure (CIAF) to assess undernutrition among children under 5 years. (DOCX)

S2 Table Classification of composite index of severe anthropometric failure (CISAF) to assess undernutrition among children under 5 years. (DOCX)
