# Supplementary material for: Determinants of aggregate anthropometric failure among children under-five years in Ethiopia: Application of multilevel mixed-effects negative binomial regression modeling
Source: PLOS Glob Public Health. 2024 Jun 4;4(6):e0003305. doi: 10.1371/journal.pgph.0003305 (PMC11149882; doi:10.1371/journal.pgph.0003305)
Supplement: S1 Table — (DOCX) [file pgph.0003305.s001.docx]

**S1_Table: Classification of composite index of anthropometric failure (CIAF) to assess undernutrition among children under 5 years**

| Group | Descriptions | Description of the levels | Wasting | Stunting | Underweight |
| --- | --- | --- | --- | --- | --- |
| A | No failure | Normal WAZ, HAZ, and WHZ | No | No | No |
| B | Wasting only | WAZ <−2SD but normal HAZ and WHZ | Yes | No | No |
| C | Wasting and underweight | WAZ and WHZ <−2 SD but HAZ normal | Yes | No | Yes |
| D | Stunting, wasting, and underweight | HAZ and WAZ and WHZ <−2SD | Yes | Yes | Yes |
| E | Stunting and underweight | HAZ and WHZ<−2SD but WAZ normal | No | Yes | Yes |
| F | Stunting only | HAZ<−2SD but normal WAZ and WHZ | No | Yes | No |
| Y | Underweight only | WHZ<−2SD but normal HAZ and WAZ | No | No | Yes |
