# Supplementary material for: Determinants of aggregate anthropometric failure among children under-five years in Ethiopia: Application of multilevel mixed-effects negative binomial regression modeling
Source: PLOS Glob Public Health. 2024 Jun 4;4(6):e0003305. doi: 10.1371/journal.pgph.0003305 (PMC11149882; doi:10.1371/journal.pgph.0003305)
Supplement: S2 Table — (DOCX) [file pgph.0003305.s002.docx]

**S2_Table: Classification of composite index of severe anthropometric failure (CISAF) to assess undernutrition among children under 5 years**

| Group | Descriptions | Severe wasting | Severe stunting | Severe underweight |
| --- | --- | --- | --- | --- |
| A | No severe failure | No | No | No |
| B | Severe wasting only | Yes | No | No |
| C | Severe wasting and severe underweight | Yes | No | Yes |
| D | Severe wasting, severe stunting and severe underweight | Yes | Yes | Yes |
| E | Severe stunting and severe underweight | No | Yes | Yes |
| F | Severe stunting only | No | Yes | No |
| G | Severe underweight only | No | No | Yes |

Note: A child was considered to be severely stunted and severely wasted and severely underweight if the z-scores of length/height-for-age, weight-for-height/length, and weight-for-age were below minus three (− 3.0) Standard Deviations (SD) (i.e., HAZ/LAZ < −3SD; WHZ/WLZ < −3SD; WAZ < −3SD) below the respective median of the World Health Organization (WHO) reference population, respectively. Children without any anthropometric failure from B to G, that is, with moderate undernutrition and/or normal children were categorized as ‘no severe failure’.
